# Perturbations both trigger and delay seizures due to generic properties of slow-fast relaxation oscillators

**DOI:** 10.1371/journal.pcbi.1008521

**Published:** 2021-03-29

**Authors:** Alberto Pérez-Cervera, Jaroslav Hlinka

**Affiliations:** 1 Department of Complex Systems, Institute of Computer Science of the Czech Academy of Sciences, Prague, Czech Republic; 2 Center for Cognition and Decision Making, Institute for Cognitive Neuroscience, National Research University Higher School of Economics, Moscow, Russia; 3 National Institute of Mental Health, Klecany, Czech Republic; Newcastle University, UNITED KINGDOM

## Abstract

The mechanisms underlying the emergence of seizures are one of the most important unresolved issues in epilepsy research. In this paper, we study how perturbations, exogenous or endogenous, may promote or delay seizure emergence. To this aim, due to the increasingly adopted view of epileptic dynamics in terms of slow-fast systems, we perform a theoretical analysis of the phase response of a generic relaxation oscillator. As relaxation oscillators are effectively bistable systems at the fast time scale, it is intuitive that perturbations of the non-seizing state with a suitable direction and amplitude may cause an immediate transition to seizure. By contrast, and perhaps less intuitively, smaller amplitude perturbations have been found to delay the spontaneous seizure initiation. By studying the isochrons of relaxation oscillators, we show that this is a generic phenomenon, with the size of such delay depending on the slow flow component. Therefore, depending on perturbation amplitudes, frequency and timing, a train of perturbations causes an occurrence increase, decrease or complete suppression of seizures. This dependence lends itself to analysis and mechanistic understanding through methods outlined in this paper. We illustrate this methodology by computing the isochrons, phase response curves and the response to perturbations in several epileptic models possessing different slow vector fields. While our theoretical results are applicable to any planar relaxation oscillator, in the motivating context of epilepsy they elucidate mechanisms of triggering and abating seizures, thus suggesting stimulation strategies with effects ranging from mere delaying to full suppression of seizures.

## Author summary

Despite its simplicity, the modelling of epileptic dynamics as a slow-fast transition between low and high activity states mediated by some slow feedback variable is a relatively novel albeit fruitful approach. This study is the first, to our knowledge, characterizing the response of such slow-fast models of epileptic brain to perturbations by computing its isochrons. Besides its numerical computation, we theoretically determine which factors shape the geometry of isochrons for planar slow-fast oscillators. As a consequence, we introduce a theoretical approach providing a clear understanding of the response of perturbations of slow-fast oscillators. Within the epilepsy context, this elucidates the origin of the contradictory role of interictal epileptiform discharges in the transition to seizure, manifested by both pro-convulsive and anti-convulsive effect depending on the amplitude, frequency and timing. More generally, this paper provides theoretical framework highlighting the role of the slow flow component on the response to perturbations in relaxation oscillators, pointing to the general phenomena in such slow-fast oscillators ubiquitous in biological systems.

## Introduction

The dynamics underlying complex processes usually involve many different time scales due to multiple constituents and their diverse interactions. When modelling such systems, the general distinction of at least two time-scales (fast and slow) is a useful and common conceptualization. Many examples of slow-fast dynamics can be found in cell modelling, ecosystems, climate or chemical reactions [1–4] and more recently in epilepsy [5], of particular interest for this paper.

Epilepsy is a chronic neurological disorder characterized by a marked shift in brain dynamics caused by an excessively active and synchronized neuronal population [6, 7]. Although several dynamical pathways have been proposed to explain the transition to seizure [8–12], in general, epileptic tissue is modelled as a system having two stable states: one corresponding to the low activity state and the other corresponding to high activity (that is to seizure) [13]. Besides external perturbations or noise, transitions between these two stable states can also be modelled considering the existence of a parameter evolving on some slow time scale. Whereas on the fast time scale the system can be seen as a bistable system, the variations of the slow parameter lead to bifurcations providing transitions between states [14].

During the last decade, there has been an increasing number of models approaching epilepsy through slow-fast time scales [15–21]. Recently, the slow-fast dynamics has been proposed to explain the role of the interictal epileptiform discharges (IEDs) in the generation of seizures [22]. The IEDs can be thought of as endogenous inputs affecting the target tissue. However, the effect of IEDs on the tissue activity is quite controversial: where some studies show that IEDs can prevent seizures [23, 24], other studies claim their seizure facilitating role [25, 26]. In the above mentioned work [22], the amplitude and frequency dependence of the effect of perturbations in a simple epilepsy model switching between seizure and non-seizure states due to a slow feedback variable, provided a conceptual reconciliation of the pro-convulsive and anti-convulsive effect of IEDs.

In this paper we elucidate this phenomenon in detail and provide theoretical foundations of this apparent perturbation effect paradox by studying the phase response of a generic relaxation oscillator. We perform this theoretical approach by means of the phase reduction [27]. In addition to simplifying the dynamics, the usage of phase reduction techniques allows the computation of its *isochrons* and *phase response curves* (PRCs), which clarify the dependence of the effect of perturbations of the oscillator on the perturbation timing, and also allows the study of possible synchronization regimes [28]. By studying a generic slow-fast system displaying relaxation oscillations we show, analytically, how the slow component of the vector field shapes their isochrons and PRCs, thus ultimately determining its response to perturbations. Therefore, our results, clarify the multifaceted effect of IEDs in epilepsy, and can be straightforwardly applied to understand the temporal dependency of perturbations over any model belonging to the wide family of models relying on slow-fast dynamics.

The paper is structured as follows. First, we present a general introduction to relaxation oscillators introducing the basic notation which will be used throughout the paper. Then, we describe the phenomenological epilepsy model and show how, through its phase analysis, we can unveil the mechanism integrating the contradictory role of IEDs in epilepsy. Next, we show, via a complete theoretical analysis, which factors determine the geometry of isochrons of planar relaxation oscillators and study the response of perturbations of relaxation oscillators. We support our theoretical findings studying the response of perturbations for a different reduced epileptor model and discuss our results in the context of epilepsy. We conclude the paper by explaining the computational techniques in the Materials and Methods section.

## Results

### Basic introduction to relaxation oscillations

**Fig 1:**
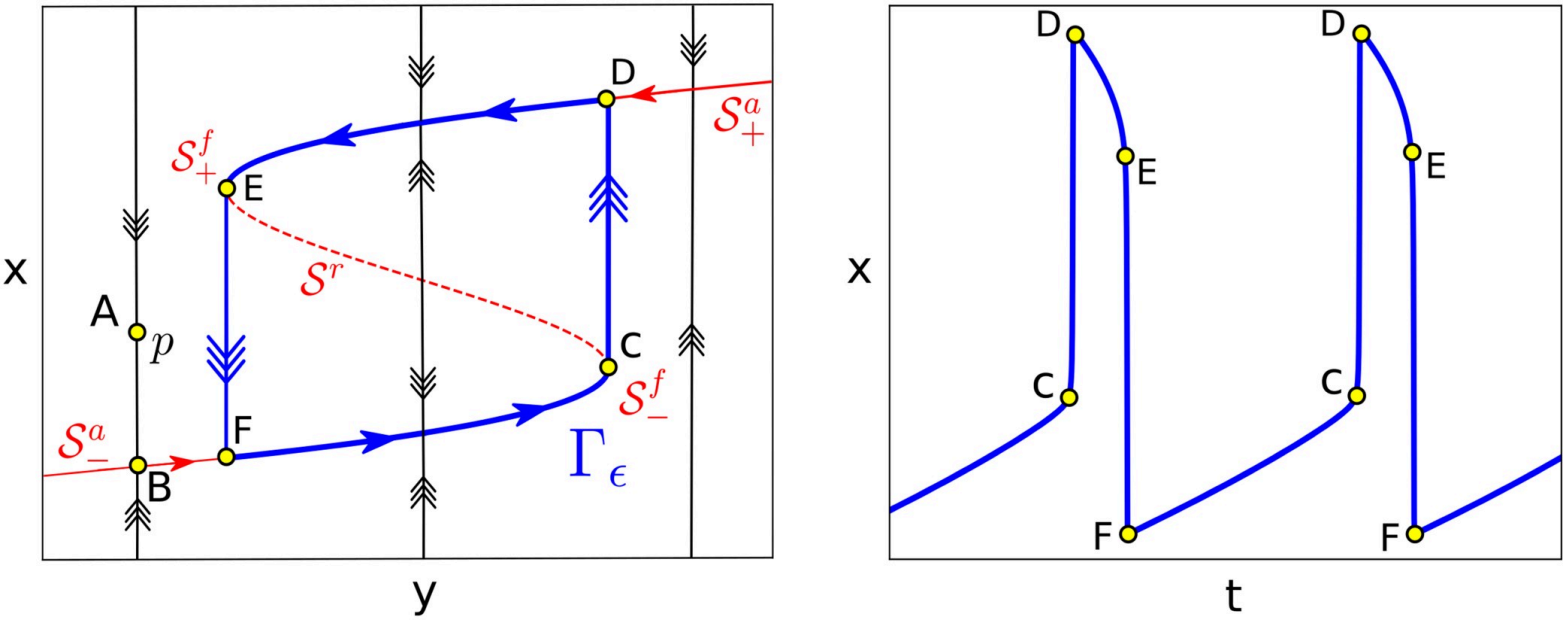
Phase space for relaxation oscillators. The slow manifold, S, is a S-shaped curve having two stable branches $S_{\pm }^{a}$ (solid red line) and one repelling $S^{r}$ (dashed red line) (see Eq (4)). Stable and unstable branches of S are separated by the fold points $S_{\pm }^{f}$. A given point, *p*, (see A) will quickly converge to the attracting branch of the slow manifold $S_{-}^{a}$ (see B). Then, it evolves along $S_{-}^{a}$ following (6) until reaching the fold point $S_{-}^{f}$ (see C) where it traverses fast to the other branch $S_{+}^{a}$ (see D). Then, following again the slow dynamics, the trajectory approaches $S_{+}^{f}$ (see E) where it goes back to $S_{-}^{a}$ (see F). Therefore, the system (1) in the singular limit (*ϵ* → 0) admits a singular periodic orbit Γ_*ϵ*_ (in blue) generating relaxation oscillations.

The main aim of this Section is to introduce the reader to the basics of slow-fast systems and in particular to relaxation oscillations. For further details we refer the reader to [29–32]. We will consider systems in this form

$$\begin{array}{c} \begin{array}{c} \dot{x}=f(x,y),\\ \dot{y}=\epsilon g(x,y),\;0\leq \epsilon \ll 1 \end{array} \end{array}$$ (1)

the flow of which will be denoted as *ϕ*_*t*_(*x*, *y*). Notice that ˙ indicates the derivative with respect to the time, *t*. As 0 ≤ *ϵ* ≪ 1, the variables *x* and *y* evolve on different time-scales, namely the fast time, *t*, and the slow time *τ* = *ϵt*. Next, we use this distinction between time-scales to illustrate how a system in the form (1) with the extra assumption of *f*(*x*, *y*) = 0 being a cubic manifold, generates a limit cycle (denoted as Γ_*ϵ*_) showing relaxation oscillations [33] (see also Fig 1). Consider a point *p* = (*x*, *y*). First, since *ϵ* ≪ 1, we can take the limit *ϵ* → 0 and approximate the dynamics of system (1) by the *layer* dynamics

$$\begin{array}{c} \begin{array}{c} \dot{x}=f(x,y),\\ \dot{y}=0. \end{array} \end{array}$$ (2)

The trajectory of *p* will initially (approximately) follow the layer dynamics in (2) so it will quickly converge to its set of equilibrium points, defined as the *slow manifold* S

$$\begin{array}{c} S=\{\left(x,y\right)\in R^{2}\;|\;f\left(x,y\right)=0\}, \end{array}$$ (3)

which in the limit *ϵ* → 0 corresponds to the nullcline ($\dot{x}=0$) of the fast variable. As we considered the slow manifold S in (3) to be cubic, that is S-shaped, it will have two fold points (given by ∂_*x*_ *f*(*x*, *y*) = 0), which we denote as $S_{+}^{f},S_{-}^{f}$ respectively, separating the repelling and attracting branches, denoted as $S^{r}$ and $S^{a}$, respectively

$$\begin{array}{c} \begin{array}{c} S^{r}=\{\left(x,y\right)\in S\;|\;\partial_{x}f\left(x,y\right)>0\},\\ S^{a}=\{\left(x,y\right)\in S\;|\;\partial_{x}f\left(x,y\right)<0\}. \end{array} \end{array}$$ (4)

Note that the attracting part of the slow manifold $S^{a}$ in fact consists of a top and bottom branch $S_{\pm }^{a}$. Once the system has approached the slow manifold, its dynamics are given by the slow variable

$$\begin{array}{c} \begin{array}{c} 0=f(x,y),\\ y^{\prime }=g(x,y), \end{array} \end{array}$$ (5)

where ′ denotes the derivative with respect to the *slow* time *τ* = *ϵt*. Furthermore, for points in S satisfying ∂_*x*_ *f*(*x*, *y*) ≠ 0 we know from the implicit function theorem that we can write a function *x* = *m*(*y*) from *f*(*x*, *y*) = 0, so we can express (5) as

$$\begin{array}{c} y^{\prime }=g\left(m\left(y\right),y\right). \end{array}$$ (6)

Therefore, once the trajectory has converged to the slow manifold, S, the *y* variable evolves following the dynamics in (6), while the *x* variable is given by *x* = *m*(*y*). So trajectories slowly move along S until reaching the fold points $S_{\pm }^{f}$. There, they become governed by the fast dynamics, leading to an almost instantaneous transition to the other stable branch. Indeed, as Fig 1 shows, this is the mechanism underlying the generation of a stable periodic orbit Γ_*ϵ*_ showing relaxation oscillations, that is, the motion over Γ_*ϵ*_ consists of the alternation of long intervals of quasi-static behaviour (corresponding to the stable branches $S_{\pm }^{a}$ of S) and almost instantaneous transitions between the branches [31].

### Phenomenological epilepsy model

As we discussed in the introduction, the mechanism of relaxation oscillations (see Fig 1) has been recently used in [22] to explain the apparent contradictory role of IEDs in epilepsy. In this work, the authors propose the following simple phenomenological epilepsy model, further referred to simply as the *phenomenor*:

$$\begin{array}{c} \begin{array}{c} \dot{v}=-\tau_{x}\left(v^{3}+v^{2}-a\right),\\ \dot{a}=\tau_{a}f\left(h-v\right), \end{array} \end{array}$$ (7)

where *v* and *a* represent the firing rate and the excitability of a neuronal population, respectively. The dynamical changes in the excitability depend on the difference between *v* and *h* through the function *f*(*x*) = (tanh(*cx*) − *a*_0_), that is, an hyperbolic tangent whose slope is given by *c*. When *v* values are below *h*, the excitability increases, whereas when *v* values exceed *h*, the excitability decreases. Hence, *h* can be thought of as a threshold. For this study, *h* = *h*_*m*_ *a* − *h*_*n*_. We keep fixed the particular set of parameters

$$\begin{array}{c} \begin{array}{c} P_{pheno}=\left\{\tau_{x}=1,\;\tau_{a}=0.001,\;c=1000,\;h_{n}=0.86,\;h_{m}=1.6,\;a_{0}=0.5\right\}, \end{array} \end{array}$$ (8)

for which the system (7) displays a limit cycle denoted as Γ_*pheno*_ with a period of *T* ≈ 508.42; although the qualitative behaviour of the model stays the same for a wide range of parameters. Indeed, as *τ*_*a*_ ≪ *τ*_*x*_ and the fast nullcline $\dot{v}=0$—which corresponds with the slow manifold S in (3)—describes a cubic curve, dynamics over Γ_*pheno*_ consists of a periodic switching between the states of low and high activity within relaxation oscillations.

**Fig 2:**
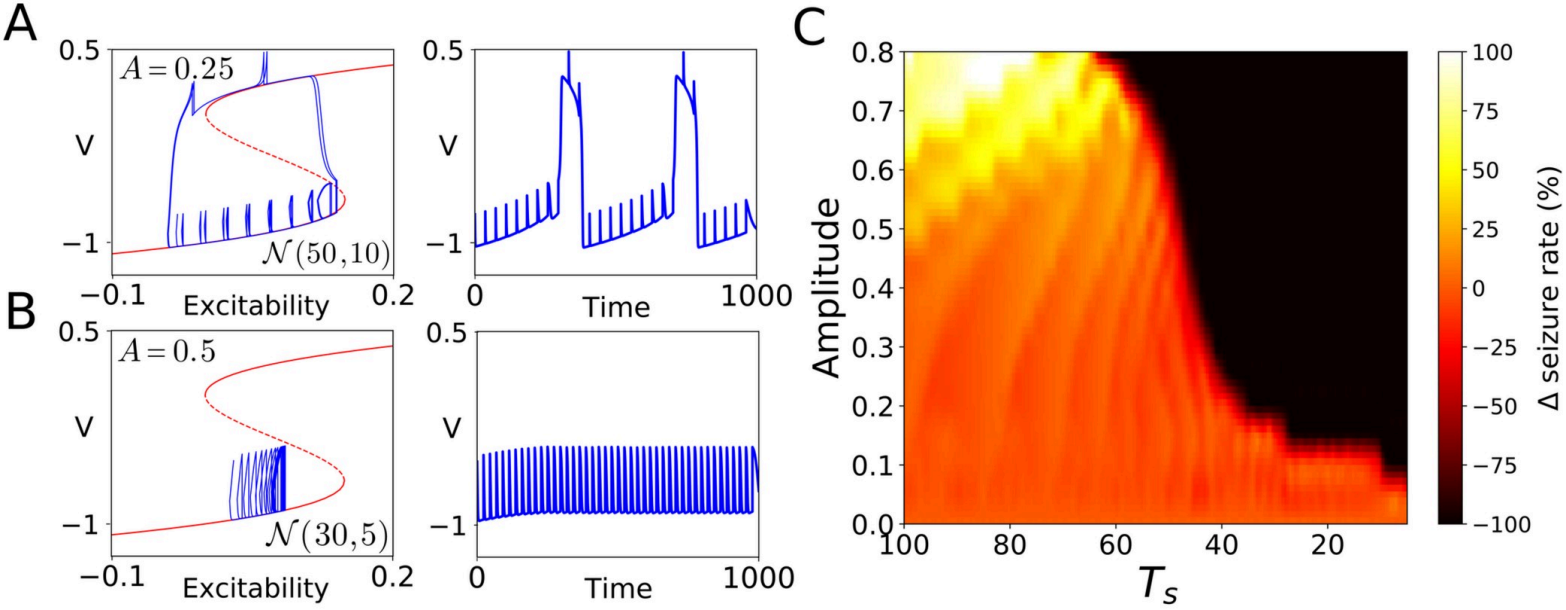
The antagonistic effect of IEDs on the transition to seizure. Panels A and B show, in red, the v-nullcline whose stable branches correspond to the stable low and high activity states of the system. The unstable part of the v-nullcline (dashed red line) separates the basin of attraction of both branches. As was illustrated in [22], Figure 5, whether the pulses make the system cross the unstable part of the v-nullcline determines the opposite nature of IEDs. For a random train with amplitude *A* = 0.25 and $t_{s}∼N\left(50,3^{2}\right)$ the system goes to seizure (panel A). By contrast, for a random train with amplitude *A* = 0.5 and $t_{s}∼N\left(30,2^{2}\right)$ the system avoids the seizure state (panel B). By plotting the change in seizure rate Δ as a function of both the amplitude, *A*, and the mean inter-perturbation interval, *T*_*s*_ (panel C), we can distinguish between pro-convulsive regimes (yellow and white areas) in which the transition is potentiated, and preventive regimes (red and black areas) in which the transition is delayed or completely suppressed. We refer the reader to Materials and Methods section for the specific details about the computation of panel C.

The following Fig 2 illustrates the mechanism proposed in [22] by which the phenomenor (7) reconciles the antagonistic role of IEDs. Consider the IEDs as a random train of pulses whose inter pulse interval distribution, *t*_*s*_, follows a normal distribution with mean value, *T*_*s*_, and standard deviation, *σ*: $t_{s}∼N\left(T_{s},\sigma^{2}\right)$. Whether or not a given perturbation causes an immediate transition to seizure depends on whether the perturbation manages to make the trajectory cross from the lower branch above the middle branch of the v-nullcline. If this happens, the trajectory rapidly converges to the upper branch, i.e. transitioning to the seizure regime. However, the response of the system dramatically changes depending on the amplitude, *A*, and mean inter pulse interval, *T*_*s*_, of IEDs (see Fig 2A and 2B).

The effect of a single pulse applied to the system, while on the lower branch, is either to keep the trajectory on the lower branch or to cause a transition to the upper branch. Therefore, the total effect of a train of pulses depends on the proportion of pulses causing transitions. Indeed, this dependence can be seen by plotting the change in the seizure rate Δ as a function of both the amplitude, *A*, and the mean inter-perturbation interval, *T*_*s*_ (Panel C).

### Phase dynamics

Oscillations correspond to attracting limit cycles whose dynamics can be described by a single variable: the phase. As we now expose, the study of the dynamics on a limit cycle by means of the phase variable provides a more intuitive and simplified view of its synchronization properties. Consider an autonomous system of ODEs

$$\begin{array}{c} \dot{z}=Z\left(z\right),\;z\in R^{d},\;d\geq 2, \end{array}$$ (9)

whose flow is denoted by *ϕ*_*t*_(*z*). Assume that *Z* is an analytic vector field and that system (9) has a *T*-periodic hyperbolic attracting limit cycle, Γ. This *T*-periodic limit cycle, Γ, can be parametrized by the phase variable *θ* = *t*/*T* as

$$\begin{array}{c} \begin{array}{c} \gamma :T≔R/Z\to R^{d}\\ \theta \mapsto \gamma (\theta ), \end{array} \end{array}$$ (10)

so that it has period 1, that is, *γ*(*θ*) = *γ*(*θ* + 1). While originally defined only on the limit cycle, the phase can be extended to the whole basin of attraction of Γ (which we will denote by W). Indeed, as we consider attracting limit cycles, any point in W converges towards Γ as time tends to infinity. Therefore, we will say that two points p,q∈W have the same asymptotic phase if

$$\begin{array}{c} \lim_{t\to \infty }\left|\phi_{t}\left(q\right)-\phi_{t}\left(p\right)\right|=0. \end{array}$$ (11)

We further define the isochron $I_{\theta }$ as the set of points having the same asymptotic phase *θ* [34], that is,

$$\begin{array}{c} I_{\theta }=\{z\in W|\;|\phi_{t}\left(z\right)-\phi_{t}\left(\gamma \left(\theta \right)\right)|=|\phi_{t}\left(z\right)-\gamma (\theta +\frac{t}{T})|\to 0\;\text{as}\;t\to \infty \}. \end{array}$$ (12)

Let us now consider the effect of an instantaneous delta-like pulse over the *T*-periodic limit cycle Γ,

$$\begin{array}{c} p\left(z,t;A\right)=\delta \left(t-t_{s}\right). \end{array}$$ (13)

**Fig 3:**
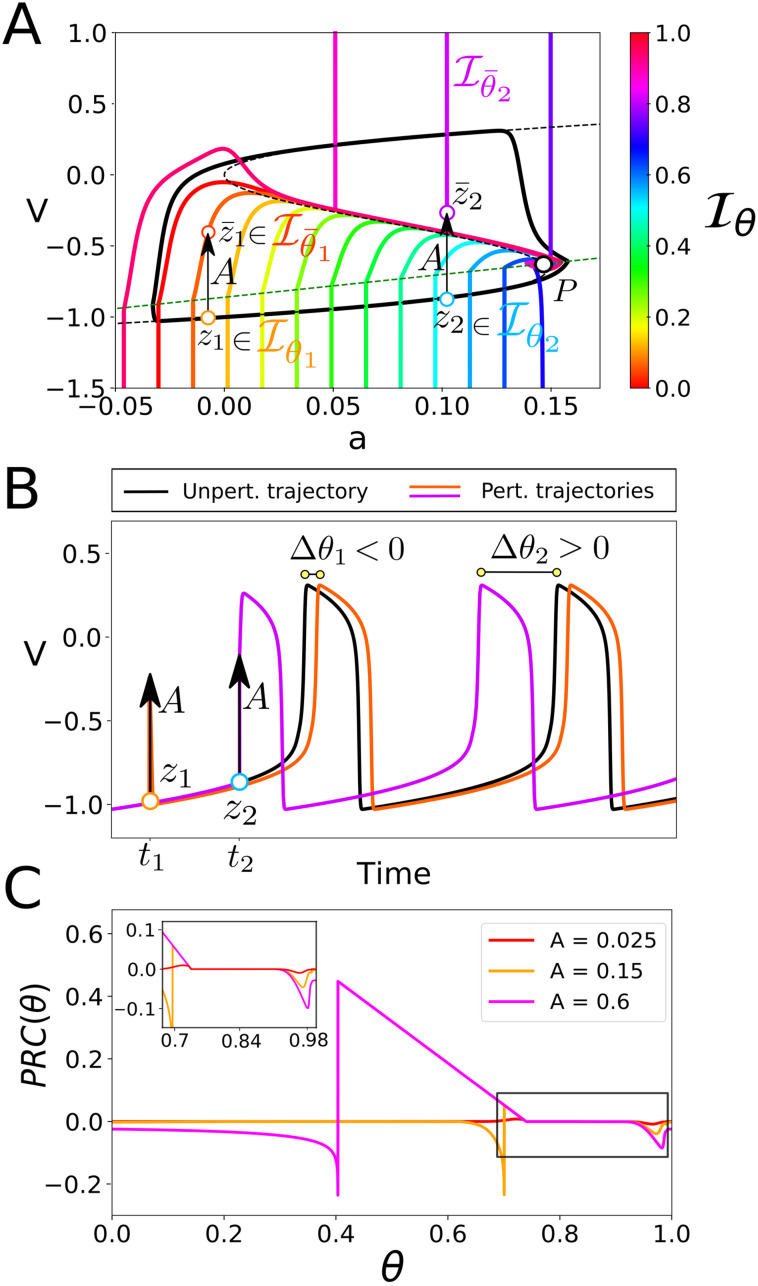
Isochrons and PRCs for the phenomenor model (7). Panel (A) shows the limit cycle Γ_*pheno*_, 16 equispaced isochrons, the *v* and *a* nullclines (dashed black and green curves, respectively) and the fixed point, *P*, at their intersection. The distribution of isochrons clarifies the time dependency of perturbations: as panel (B) shows, a pulse of amplitude *A* applied at a time *t*_1_ (*t*_2_) causes a negative (positive) phase shift, delaying (promoting) the transition to seizure. This time dependency can be directly inferred from panel (A): a pulse of amplitude *A* applied at a point on the cycle *z*_1_ = *γ*(*θ*_1_) = *γ*(*t*_1_/*T*) (*z*_2_ = *γ*(*θ*_2_) = *γ*(*t*_2_/*T*)) displaces the trajectory to a point ${\bar{z}}_{1}\in I_{{\bar{\theta }}_{1}}$ (${\bar{z}}_{2}\in I_{{\bar{\theta }}_{2}}$). Since ${\bar{\theta }}_{1}<\theta_{1}$ (${\bar{\theta }}_{2}>\theta_{2}$) the perturbation causes a phase shift $\Delta \theta_{1}={\bar{\theta }}_{1}-\theta_{1}<0$ ($\Delta \theta_{2}={\bar{\theta }}_{2}-\theta_{2}>0$) delaying (advancing) the phase of the oscillator. The panel (C) shows the PRCs for the phenomenor for positive voltage pulses of different amplitudes summarising the timing (phasic) effect of a given perturbation.

It is clear that the perturbation will just change the trajectory from one point *z* to another point $\bar{z}$. As we illustrate in Fig 3, since the isochrons foliate the whole basin of attraction W of Γ, we can say that the perturbation moved the trajectory from one isochron $I_{\theta }$ to another isochron $I_{\bar{\theta }}$, thus causing a phase shift $\Delta \theta =\bar{\theta }-\theta$. However, the phase shift will depend on the amplitude of the pulse and on the phase at which it was applied. This dependency is captured by the Phase Response Curves (PRCs). They are calculated by applying the same pulse to the limit cycle at different phases and registering how much the phase is advanced (or delayed). Let *z* = *γ*(*θ*) be a point on the limit cycle Γ. If we consider an instantaneous pulse as (13), it is clear that it will move *z* to $\bar{z}=z$ + Δ*z*. Thus, the PRC is defined as

$$\begin{array}{c} PRC\left(A,\theta \right)=\bar{\theta }-\theta . \end{array}$$ (14)

As Fig 3A shows, the isochrons $I_{\theta }$ of Γ_*pheno*_ portray the distribution of phases along the basin of attraction W. Whereas the isochrons for the upper branch of the cycle are almost vertical, the isochrons for the lower branch of the cycle show a more interesting geometry: they start vertical until crossing the *a*-nullcline, when they all bend. The shape of the PRCs as the amplitude, *A*, of the pulse increases is determined by this particular geometry of the isochrons. Since there is an almost constant distance of 0.1 between the lower branch of Γ_*pheno*_ and the slow nullcline $\dot{a}=0$, we can distinguish between two cases. For perturbations of *A* < 0.1, the perturbed trajectories only reach the part of isochrons consisting in almost vertical lines. Therefore, the corresponding phase shift Δ*θ* for perturbations on the upper and lower branches of Γ_*pheno*_ is almost negligible. Hence, the PRC for these phases will be close to zero. Indeed, only in the vicinity of the fold points $S_{\pm }^{f}$ the PRCs will show larger values (see zoom window in Fig 3C).

By contrast, for perturbations of *A* > 0.1, the change on the geometry of isochrons for points on the lower branch remarkably changes the shape of PRCs. Perturbations on the lower branch will have a delaying effect unless they bring trajectories above the middle branch of the v-nullcline—which corresponds with $S^{r}$ in (4)—so they advance phase (see points *z*_1_ and *z*_2_ at Fig 3A and 3B illustrating delaying and advancing effects, respectively). The delaying or advancing effect of a given pulse of amplitude, *A*, is delimited across a discontinuity for its corresponding PRC at the exact phase θ* for which $\gamma \left(\theta^{*}\right)+A\in S^{r}$ (see Fig 3C).

The isochrons and PRCs computed for the phenomenor provide insight about how the combination of both the amplitude, *A*, and the mean inter pulse interval, *T*_*s*_, generate the different seizure propensity regimes in Fig 2C. As isochrons in Fig 3 show, unless they bring trajectories above $S^{r}$, the effect of positive voltage pulses at a point, *z* = *γ*(*θ*), on the lower branch is to cause a delay Δ*θ* < 0. However, for large enough mean inter-pulse intervals *T*_*s*_, although perturbations delay the system, they are not frequent enough to stop it from eventually transitioning to seizure (see Fig 2A). Moreover, larger pulses are able to cause the trajectory to cross the v-nullcline earlier through the cycle (way before the fold point). Thus, the larger the amplitude of the pulse, the more common are these transitions. By contrast, for small enough inter-pulse intervals, *T*_*s*_, the transition to seizure can be delayed or even stopped across the accumulation of the delays caused by each single pulse (see Fig 2B). Thus we can conclude that the mechanism underlying the description of the phenomenor of the role of IEDs, relies on the one hand on its cubic v-nullcline structure, allowing for relaxation oscillations and on the other hand on the prevalence of delays for positive perturbations at points on the lower branch not crossing the middle branch of the v-nullcline.

### Phase analysis of relaxation oscillators

As explained in the previous Section, the accurate description of the role of IEDs provided by the phenomenor is based on the prevalence of delays for perturbations in the ‘non-epileptic’ state, i.e. on the bottom branch of the cycle Γ_*pheno*_. Since this determining feature of the model—the prevalence of delays—is based on the bending in a particular direction of the isochrons, we aim to identify which elements in the model are key to cause this particular isochron geometry. As we show next, we perform this identification by taking advantage of the dynamical properties underlying any relaxation oscillator.

#### The O(1) geometry of isochrons $I_{\theta }$

Next, we discuss some generalities shaping the isochrons of planar relaxation oscillators. To begin, it is worth recalling that if two points $\bar{z}\in W$ and *z* = *γ*(*θ*) belong to the same isochron, $I_{\theta }$, they have to meet at the same point of the cycle after a large enough time, *t* (see Eq 12). For this reason, the determination of the shape of isochrons requires to study the converging dynamics towards Γ_*ϵ*_ which we recall consist of trajectories covering O(1) distances in the fast direction and O(ϵ) distances in the slow direction.

Since we aim to study the isochrons for relaxation oscillators, we can take advantage of the time-scale separation to be more precise concerning this convergence. Consider a point $\bar{z}\in W$. In a first approximation one can assume that the convergence of $\bar{z}$ is achieved simply following the layer dynamics (2). If that was the case, since the layer dynamics consider the variable *y* as frozen, the isochrons will always be lines of *y* constant that we denote as $F_{y}$. However, for correctly determining the shape of isochrons, we have to take into account that neither the convergence towards the limit cycle Γ_*ϵ*_ is instantaneous nor the dynamics on *y* during convergence are negligible. As a result, the isochrons are expected to be O(ϵ) corrections of $F_{y}$. Indeed, it is worth to note that generalities determining the sign of those O(ϵ) corrections will explain the prevalence of delaying (or advancing) effects of delta-like pulses in the fast direction.

Regarding the time needed for solutions to converge to the limit cycle, although the convergence towards a normally hyperbolic attracting limit cycle is ensured [35], for the case of slow-fast dynamics we can give even more details about this convergence by means of Tikhonov’s theorem [36] (see also [37, 38]). Roughly speaking, Tikhonov’s theorem states that after a time $t_{h}=O\left(\epsilon |\log\epsilon |\right)$, all orbits starting in a neighbourhood O(1) of the slow manifold S will have reached a neighbourhood of O(ϵ) of S.

Once we know the time, $t_{h}=O\left(\epsilon |\log\epsilon |\right)$, needed to converge, we can compute the motion of the converging point $\bar{z}\in W$ in the slow direction. The travelled distance in the *y* direction by $\bar{z}$ to approach a O(ϵ) neighbourhood of Γ_*ϵ*_ is given by

$$\begin{array}{c} \Delta \bar{y}=y_{th}-\bar{y}=\epsilon \int_{0}^{t_{h}}g\left(\phi_{t}\left(\bar{x},\bar{y}\right)\right)dt=\epsilon \int_{0}^{t_{h}}g\left(\varphi_{t}\left(\bar{x}\right),\bar{y}\right)dt+O\left(\epsilon^{2}\right), \end{array}$$ (15)

where $\varphi_{t}\left(\bar{x}\right)$ refers to the solution of the layer system (2). During the time, *t*_*h*_, needed to converge, the point *z* = *γ*(*θ*) on the limit cycle has travelled a distance Δ*y* given by

$$\begin{array}{c} \Delta y=y_{th}-y=\epsilon \int_{0}^{t_{h}}g\left(\phi_{t}\left(x,y\right)\right)dt=\epsilon \int_{0}^{t_{h}}g\left(m\left(\gamma^{y}\left(t/T\right)\right),\gamma^{y}\left(t/T\right)\right)dt, \end{array}$$ (16)

where in the second equality we utilize the fact that for points on the slow manifold we can use Eq (6).

**Fig 4:**
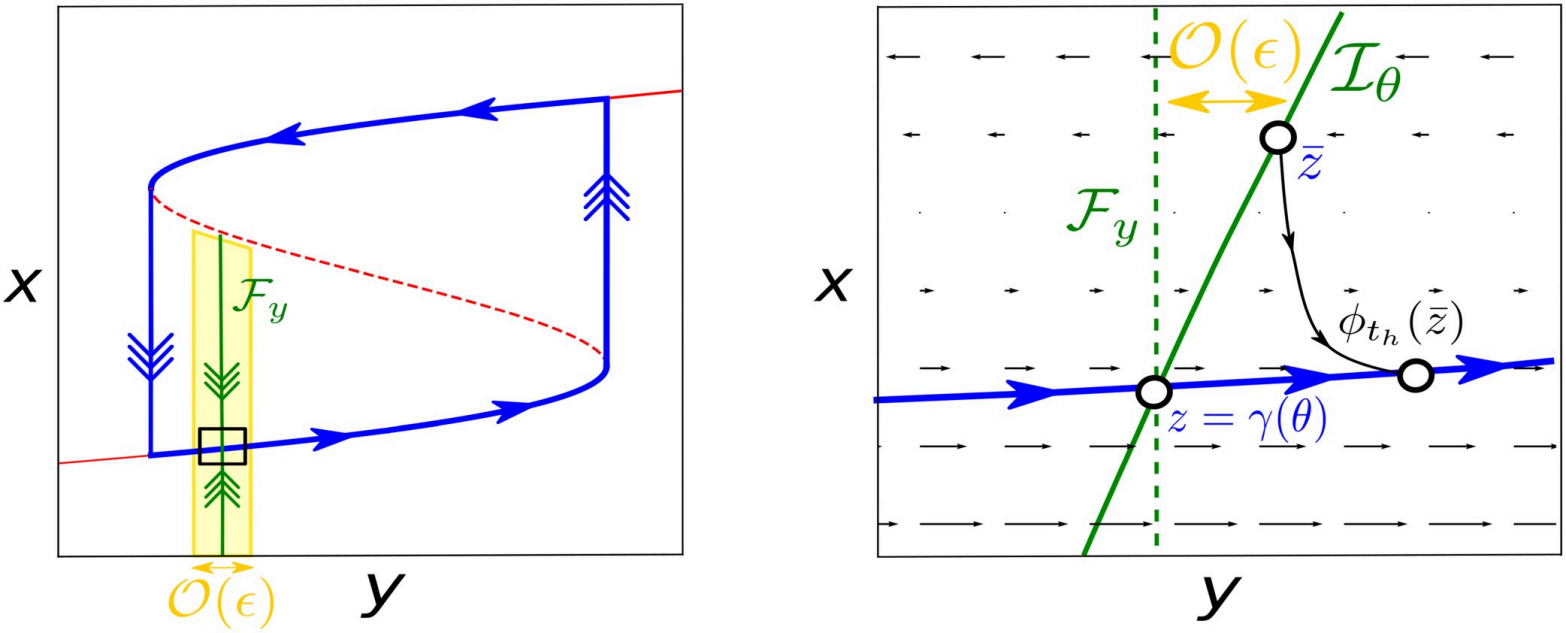
The slow vector field shapes the isochrons for relaxation oscillators. In the limit *ϵ* → 0 isochrons are lines of *y* constant denoted by $F_{y}$. However, since *ϵ* ≠ 0 but small, the isochrons are O(ϵ) perturbations of $F_{y}$. As we show in the right panel, the sign of the O(ϵ) corrections depends on the difference of speeds between the converging point $\bar{z}$ and the base point *z* during the convergence time *t*_*h*_. In this case, to approach Γ_*ϵ*_, $\bar{z}$ has to cross layers of *x* whose values are smaller than the ones surrounding Γ_*ϵ*_. For this reason $\bar{z}$ travels slower than *z*. Since $\bar{z}$ and *z* have to meet after a time *t*_*h*_ at the same point on Γ_*ϵ*_, but $\bar{z}$ travels slower than *z*, then $\bar{z}$ needs to travel a short distance. This determines the sign of the O(ϵ) correction. Furthermore, if the slow vector field is monotonous along the fast direction, the farther the point $\bar{z}$, the slower (faster) it travels, so the slope of the isochrons will have the same sign for all the points $\bar{z}\in I_{\theta }$ satisfying fast convergence, thus determining the effect of perturbations in the fast direction.

Now, let us illustrate how the difference between the distances travelled by the base point and the converging point, $\Delta y-\Delta \bar{y}$, will determine the sign of the O(ϵ) correction for isochron $I_{\theta }$ at the point $\bar{z}$. If we write the expression for $\Delta y-\Delta \bar{y}$:

$$\begin{array}{c} \Delta y-\Delta \bar{y}=\epsilon \int_{0}^{t_{h}}g\left(m\left(\gamma^{y}\left(t/T\right)\right),\gamma^{y}\left(t/T\right)\right)dt-\epsilon \int_{0}^{t_{h}}g\left(\varphi_{t}\left(\bar{x}\right),\bar{y}\right)dt=O\left(\epsilon \right), \end{array}$$ (17)

we can see that the difference $\Delta y-\Delta \bar{y}$ is directly determined by the difference between the speeds in the slow direction for the base *z* and converging $\bar{z}$ points during the time $t_{h}=O\left(\epsilon |\log\epsilon |\right)$ needed for approaching Γ_*ϵ*_. Basically, since both points *z* and $\bar{z}$ have to meet at the same point after the same time, the one travelling slower, needs to travel less distance. The difference, $\Delta y-\Delta \bar{y}$, corresponds exactly to the O(ϵ) correction to $F_{y}$ (see Fig 4).

However, at the moment we have a local argument just justifying the shape of isochrons for a given point $\bar{z}\in W$. Nevertheless, we can globalize this argument by assuming some conditions for *g*(*x*, *y*). In particular, as we show now, if the slow vector field *g*(*x*, *y*) is monotonous in the fast direction, then the slope of the isochrons will have the same sign for all the points $\bar{z}\in I_{\theta }$ satisfying fast convergence.

The asymptotic phase defined in (11) allows to assign a phase to any point z∈W, by defining the following function Θ(*z*)

$$\begin{array}{l} \Theta :\Omega \subset {\mathbb{R}}^{2}\to T=[0,1),\\ \;z\mapsto \Theta (z)=\theta \;\text{if}\;z\in I_{\theta }, \end{array}$$ (18)

whose level curves indeed correspond to the isochrons. Let us assume we can invert Θ(*x*, *y*), so we can define the following function

$$\begin{array}{c} \begin{array}{c} I_{\theta }\left(x\right):R\to R,\\ \;\;\;\;\;x\mapsto I_{\theta }\left(x\right)=y\;\;\;\;\text{for}\;\Theta \left(x,y\right)=\theta =const. \end{array} \end{array}$$ (19)

The slope of isochron $I_{\theta }$, which we denote by $K_{\theta }$, is then given by

$$\begin{array}{c} K_{\theta }=\partial_{x}\;I_{\theta }\left(x\right), \end{array}$$ (20)

as we also have

$$\begin{array}{c} I_{\theta }\left(\bar{x}\right)=\bar{y}=\Delta y-\Delta \bar{y}+y, \end{array}$$ (21)

we can write the following expression for the slope $K_{\theta }$

$$\begin{array}{c} K_{\theta }=\partial_{x}I_{\theta }\left(x\right)=\partial_{x}(\Delta y-\Delta \bar{y}). \end{array}$$ (22)

As the term $\Delta y-\Delta \bar{y}$ can be written in integral form (see Eq (17)), the slope $K_{\theta }$, can be evaluated as the derivative of the difference of two sums (integrals)

$$\begin{array}{c} K_{\theta }=\partial_{x}(\epsilon \int_{0}^{t_{h}}g\left(m\left(\gamma^{y}\left(t/T\right)\right),\gamma^{y}\left(t/T\right)\right)dt-\epsilon \int_{0}^{t_{h}}g\left(\varphi_{t}\left(\bar{x}\right),\bar{y}\right)dt). \end{array}$$ (23)

As we see, assuming that the vector field *g*(*x*, *y*) is strictly increasing (decreasing) function with *x* it is easy to discuss the sign of $K_{\theta }$. If the trajectory followed by the approaching point, satisfies $g\left(\bar{x}\left(t_{0}\right),\bar{y}\left(t_{0}\right)\right)<g\left(\bar{x}\left(t_{0}+\delta t\right),\bar{y}\left(t_{0}+\delta t\right)\right)$ for 0 < *δt* ≤ *t*_*h*_, then, the second integral will be smaller than the first one. Since this difference will increase with $\bar{x}$, then $K_{\theta }>0\;\forall \;\bar{x}>\gamma^{x}\left(t/T\right)$. Furthermore, the larger the changes in *g*(*x*, *y*), the larger the slope. We remark that in the case $g\left(\bar{x}\left(t_{0}\right),\bar{y}\left(t_{0}\right)\right)>g\left(\bar{x}\left(t_{0}+\delta t\right),\bar{y}\left(t_{0}+\delta t\right)\right)$, we can argue identically to obtain $K_{\theta }<0\;\forall \;\bar{x}>\gamma^{x}\left(t/T\right)$.

In conclusion, we have illustrated the relationship between geometry of isochrons for relaxation oscillations and the slow vector field. First, we have shown how the tilt of the isochron $I_{\theta }$ at a given point $\bar{z}$ depends on the difference of speeds between $\bar{z}$ and the base point *z* during convergence. Furthermore, we showed that if the monotonicity of the vector field does not change, the tilt of the isochrons does not change sign as well.

**Fig 5:**
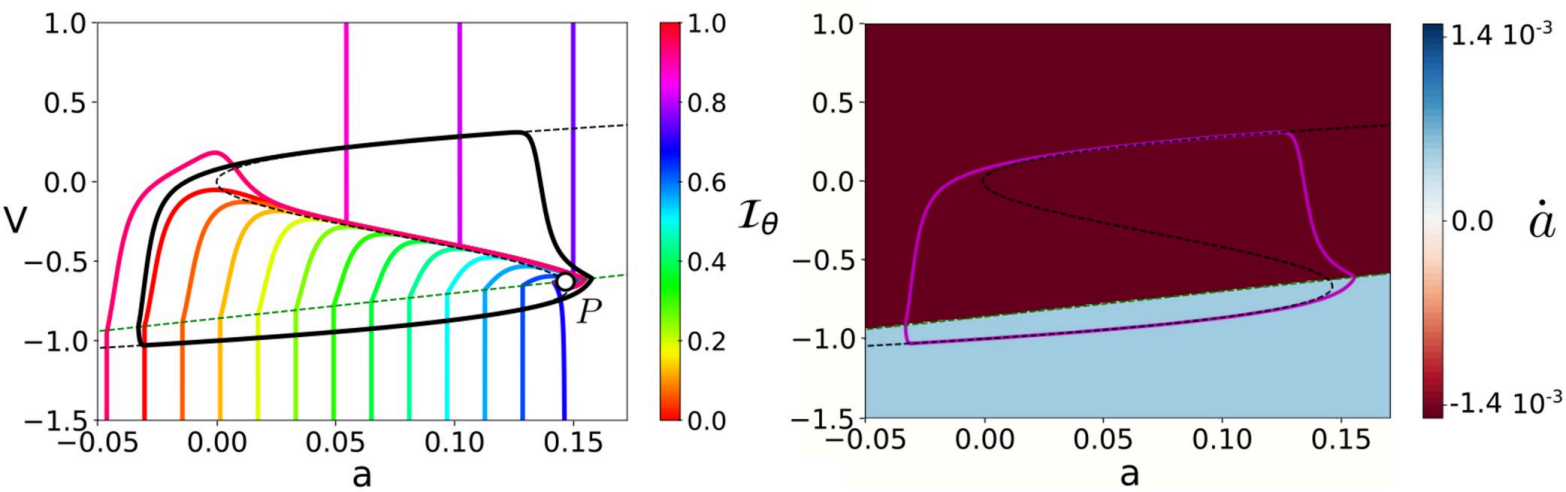
Relationship between the curvature of isochrons and the values for the slow vector field. For the phenomenological epilepsy model (7) with the set of parameters $P_{pheno}$ in (8) the figure shows: (A) Limit cycle Γ_*pheno*_ and its isochrons $I_{\theta }$ (left). (B) Values of the slow vector field (corresponding to $\dot{a}$ in (7)) for points z∈W.

We can illustrate these theoretical results by revisiting the isochrons for the phenomenor. As Fig 5 shows, the parameters $P_{pheno}$ in (8) were chosen so that the tanh in (7) acts almost as a step function. As a result, the speed in the slow direction dramatically changes when crossing the slow nullcline. Since there are almost no differences between speeds for points below the slow nullcline, the isochrons are almost vertical. By contrast, this large difference of speeds once the slow nullcline is crossed, results in a remarkable bending of the isochrons for points on the lower branch of Γ_*pheno*_.

#### PRCs

**Fig 6:**
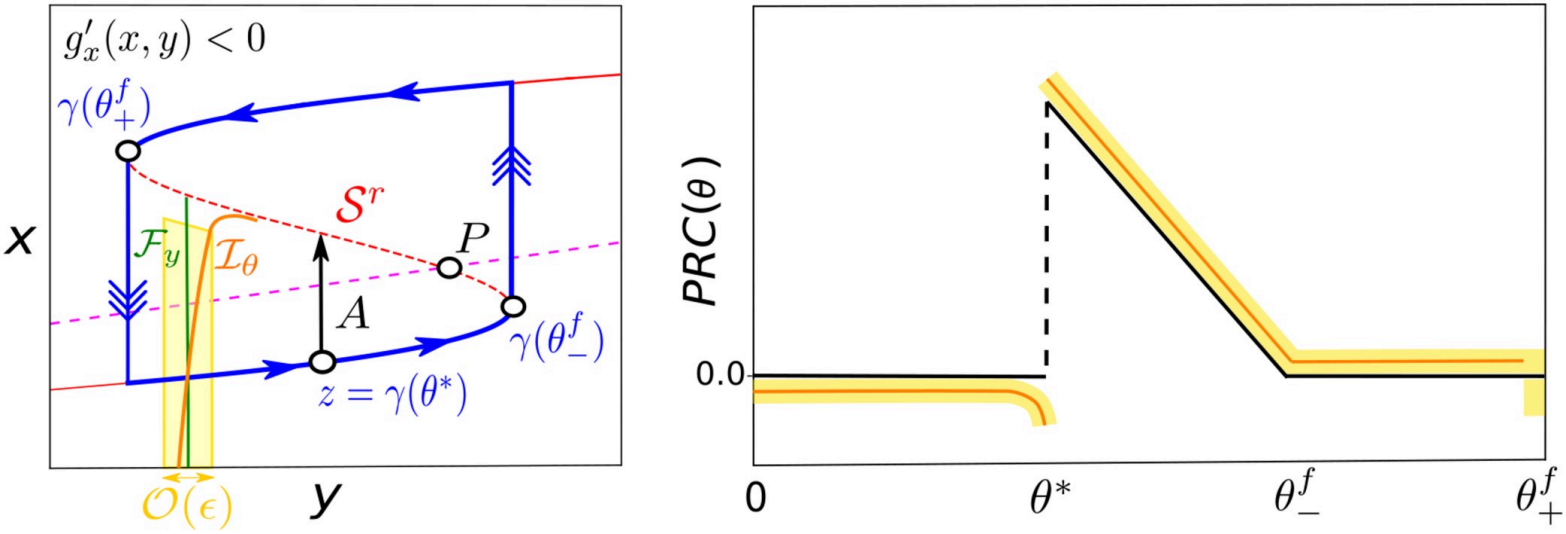
PRC of pulses *A* > 0 for relaxation oscillators. Next we sketch the PRCs for pulses of amplitude *A* > 0 for the case $g_{x}^{\prime }\left(x,y\right)<0$. For phases *θ* < θ*, where θ* satisfies $\gamma \left(\theta^{*}\right)+A\in S^{r}$, due to the slope of isochrons the effect of the pulses will be to delay trajectories. Since isochrons approach the unstable point *P* through $S^{r}$, the closer the phase *θ* to θ*, the larger the bending of the isochrons and thus the larger the corresponding delay value. For phases $\theta^{*}<\theta <\theta_{-}^{f}$, there is an advancement proportional to the fraction of cycle skipped. This prevalence of advancements is also seen for points in the upper branch. For phases in a neighbourhood of the fold point $\theta_{-}^{f}$, we expect a transition between advancement and delays not drawn because our analysis is only valid for normally hyperbolic points.

Since the shape of PRCs is determined by the geometry of isochrons, next we discuss the extensions of our previous analysis of isochrons to PRCs. First, we can consider the limit *ϵ* → 0. In this case the isochrons would be vertical lines. Therefore, for points in the lower branch, unless the pulse brings trajectories above the mid-branch $S^{r}$ of the slow nullcline, its corresponding phase shift will be zero. For those points going to the other branch, the phase shift will be proportional to the skipped segment of the cycle, thus generating the characteristic shape of PRCs for relaxation oscillators [39] (see black curve in Fig 6 right). However, our knowledge of the geometry of isochrons can extend this result. Without loss of generality we discuss the case $g_{x}^{\prime }\left(x,y\right)<0$. In this case, we know that perturbations acting over points on the lower branch not crossing $S^{r}$ will delay the system (see Fig 4). As a consequence, the PRC will have negative values for all the phases *θ* in the lower branch such that *θ* < θ* where $\gamma \left(\theta^{*}\right)+A\in S^{r}$. Although the particular shape of the delaying segment of the PRC will depend on the particular slow vector field chosen, in general, we expect the crossing of the slow and fast nullclines to generate a single unstable fixed point (denoted by *P*) inside Γ_*ϵ*_. It is worth to mention that since isochrons will approach *P* through $S^{r}$ [40], we expect the bending of a particular isochron to increase as it approaches $S^{r}$. As a consequence, we expect the maximal delay values of a PRC to concentrate near the critical phase θ*. Finally, if we consider perturbations over points in the upper branch, arguing similarly as in Fig 4, we can conclude that the effect of pulses of positive amplitude is to advance trajectories.

#### Phase locking

So far we have theoretically identified the factors shaping the isochrons for relaxation oscillators. Furthermore, we have discussed how the particular geometry of the isochrons for relaxation oscillators is reflected in the corresponding PRCs. Next, we aim to continue extending our theoretical approach to determine generalities underlying the mechanism by which external perturbations suppress the original oscillatory dynamics. We recall that, in the particular case of epilepsy, we are studying the suppression of the original oscillation through the perturbation-triggered delays which cause the system to remain in the lower activity state and thus to prevent the transition to seizure; one mechanism for this is the existence of stable phase-locked solutions of the perturbed system.

A delta-like pulse of amplitude, *A*, reaching the cycle at a phase, *θ*, will map it to a new phase *f*_*A*_(*θ*) = *θ*_*new*_, where the map *f*_*A*_(*θ*) writes as

$$\begin{array}{c} f_{A}\left(\theta \right)=\theta +PRC\left(A,\theta \right). \end{array}$$ (24)

If the perturbation was a train of periodic pulses with an inter stimulus interval given by *T*_*s*_, we can describe the phase dynamics of the system by

$$\begin{array}{c} \theta_{i+1}=f_{A}\left(\theta_{i}\right)+\frac{T_{s}}{T}=\theta_{i}+PRC\left(A,\theta_{i}\right)+\frac{T_{s}}{T}, \end{array}$$ (25)

where *θ*_0_ = *θ*. The fixed points of the above map (25), which are given by

$$\begin{array}{c} PRC\left(\theta ,A\right)=-\frac{T_{s}}{T}, \end{array}$$ (26)

correspond to the phase locking states of the system.

In the following, we shall use Eq (26) to find the phase-locked solutions that prevent seizures. Note that not all the phase-locked states predicted by Eq (26) prevent seizures. For example, consider a train of pulses whose inter stimulus interval is *T*_*s*_ ≈ *T*. Since it is forcing the system with an almost resonant frequency, the system entrains to it. However, that particular phase-locked state will not prevent seizures since the system will essentially perform one full oscillation before the next perturbation occurs.

**Fig 7:**
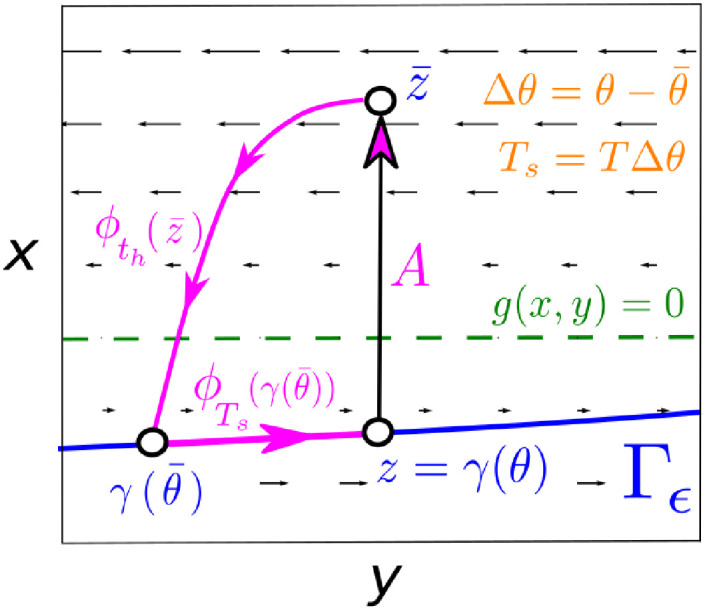
Mechanism preventing the emergence of seizures. To suppress the original oscillation and keep the system in the lower branch of Γ_*ϵ*_ the amplitude *A* of the pulse has to be large enough so besides causing a delay Δ*θ*, it displaces trajectories above enough the slow-nullcline so the distance travelled in the negative direction overcomes the distance travelled in the positive direction, thus causing a negative net displacement. The locking appears by repeating this mechanism after *T*_*s*_ = *T*Δ*θ* intervals so the new pulse always hits the system at the same initial point.

The particular phase-locked solution that *does* prevent seizures is sketched in Fig 7. Consider a pulse displacing a point *z* = *γ*(*θ*) to $\bar{z}$. If we denote by *t*_*h*_ the time that $\bar{z}$ needs to approach Γ_*ϵ*_, we need $\phi_{t_{h}}\left(\bar{z}\right)=\gamma \left(\bar{\theta }\right)$ with $\bar{\theta }<\theta$. That is, we need the perturbed trajectory to reach the cycle at a previous phase. Assuming fast convergence, we can write

$$\begin{array}{c} \gamma^{y}\left(\bar{\theta }\right)-\gamma^{y}\left(\theta \right)=\epsilon \int_{0}^{t_{h}}g\left(\bar{z}\right)dt. \end{array}$$ (27)

Since we need $\bar{\theta }<\theta$ as a necessary condition for phase locking, then, if we assume without loss of generality that the motion over Γ_*ϵ*_ is counter-clockwise, the above integral has to be negative. For that to happen, the perturbation has to necessarily send trajectories above the slow nullcline. Indeed, if we denote by t* the time needed to cross the slow nullcline, then, the particular class of locking we are interested in has to satisfy

$$\begin{array}{c} \gamma^{y}\left(\bar{\theta }\right)-\gamma^{y}\left(\theta \right)=\epsilon \int_{0}^{t_{h}}g\left(\bar{z}\right)dt=\epsilon \int_{0}^{t^{*}}g\left(\phi_{t}\left(\bar{z}\right)\right)dt+\epsilon \int_{t^{*}}^{t_{h}}g\left(\phi_{\left(t^{*}+t\right)}\left(\bar{z}\right)\right)dt<0. \end{array}$$ (28)

Since the first integral is negative and the second is positive, above Eq (28) shows that the appearance of phase locking requires the perturbed trajectories to be sent to a point such that the distance travelled during convergence in the negative direction overcomes the distance travelled in the positive direction, so the total displacement is negative. Then there is a time *T*_*s*_ = −*T*Δ*θ* > 0 (with $\Delta \theta =\bar{\theta }-\theta$) for which the next pulse will kick the system at the same initial point *z* = *γ*(*θ*) (see Fig 7). The repetition of this process keeps the trajectory on the lower branch, and prevents the seizure emergence by suppressing the original oscillatory dynamics. Importantly, we highlight the strong influence of the slow vector field on the appearance of this locking mechanism. Indeed, the smaller the distance between the slow nullcline and the lower branch, the smaller the amplitude of perturbations needed for locking the system.

**Fig 8:**
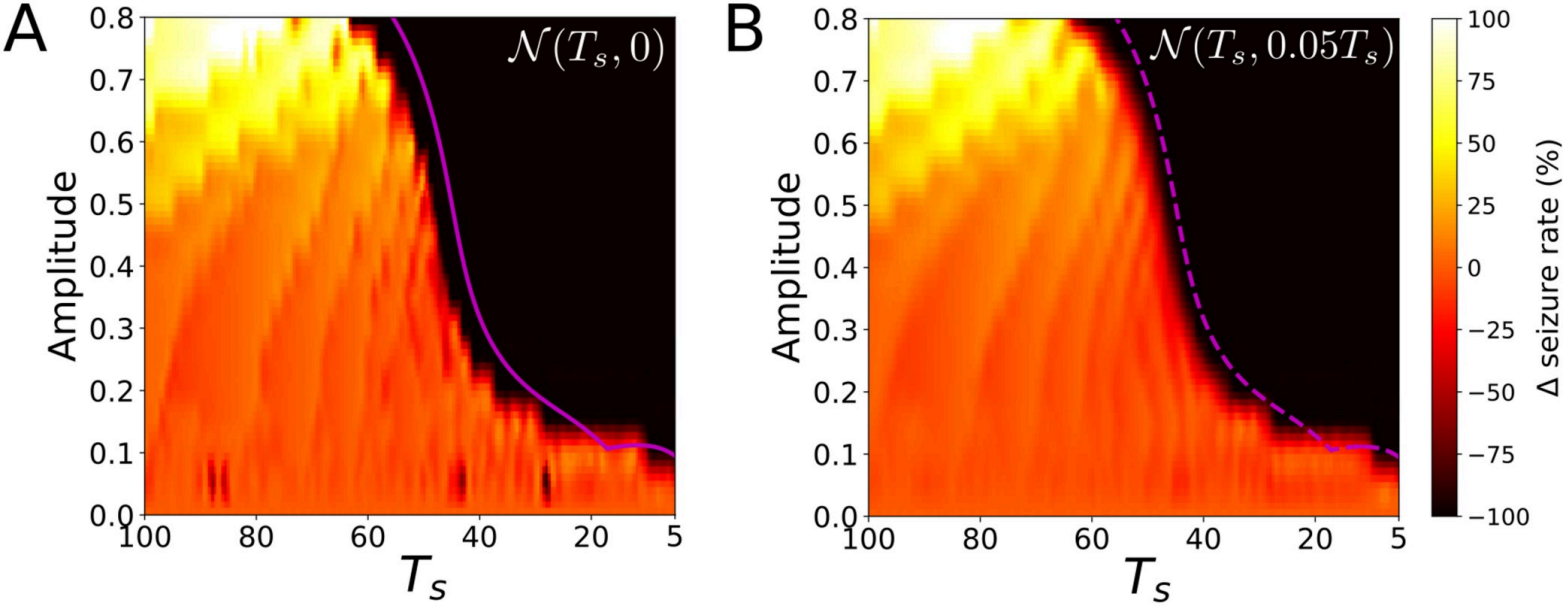
Response of perturbations for the phenomenor. We plot the change in the seizure rate Δ for a random train of pulses following a Gaussian distribution of mean time *T*_*s*_ and standard deviation *σ* denoted as $N(T_{s},\sigma^{2})$. Panels (A) and (B) correspond to the deterministic periodic case $N(T_{s},0)$ and to the random case $N(T_{s},0.05T_{s})$, respectively. For panel (A) we plot a purple solid line corresponding to the bifurcation of the phase map (25). We plot the same curve as a dashed purple curve in panel (B) illustrating the resilience of the deterministic phase-locked states to noise. For the specific details about the numerical computation of this Figure, we refer the reader to Materials and Methods section.

We can check the validity of this result by revisiting the results for the phenomenor. Fig 8A shows the relative seizure rate increase Δ for a *T*_*s*_ periodic train of pulses. We can see how the locking preventing the transition to seizure starts for values $\bar{A}\approx 0.1$, which is the approximate distance between the lower branch and the slow-nullcline. Furthermore, for a fixed amplitude $A>\bar{A}$, if we consider the maximum delay value (denoted by Δθ*) of the corresponding PRC and compute the inter pulse interval value given by $T_{s}^{*}=T\Delta \theta^{*}$, it is clear that for inter-pulse intervals $T_{s}>T_{s}^{*}$, the system is likely to transition to seizure because the delays are not large enough to stop the system. Therefore, we expect the pair $\left(A,T_{s}^{*}\right)$ to delimit the locking regime. By computing the PRCs for all the amplitude values satisfying $A>\bar{A}$, we can calculate the corresponding $T_{s}^{*}$ values and thus generate a curve in the (*A*, *T*_*s*_) space—which indeed corresponds with the bifurcation curve of the map (25)—showing a nice agreement with the boundaries of the locking area (see purple line in Fig 8A).

Results for the random case in Fig 8B can be interpreted by means of the periodic case. The random dynamics can be computed as well by using a similar map to (25) but substituting *T*_*s*_ for *t*_*s*_ values in the distribution $t_{s}∼N\left(T_{s},\sigma^{2}\right)$. In this case, the system does not ‘lock’ in the same way the deterministic system does, that is through fixed points in Eq (26). However, one might try to interpret random dynamics by means of the periodic case. As we explained for the deterministic case, the maximum delay value Δθ* of the PRC allows to compute a characteristic value of $T_{s}^{*}$, such that perturbations $t_{s}>T_{s}^{*}$ will lead to seizures. Therefore, the robustness of the deterministic locking states to noise decreases, as the probability of *t*_*s*_ being above $T_{s}^{*}$ increases, which happens with increasing the width *σ* of the inter pulse distribution.

### Epileptor model

Apart from long standing detailed epilepsy models [41], a recent successful phenomenological model of epileptic dynamics is the epileptor model [17]. This model consists of 5 differential equations (4 fast and 1 slow) so it can not only display a wide range of dynamical regimes explaining many different pathways to seizure [42], but importantly it also contains a phenomenological yet explicit deterministic mechanism for spontaneous switching between seizing and non-seizing regime. In order to show the generality of the results derived from our theoretical approach and to demonstrate their consequences in models of epilepsy, we will study the following 2D reduction of the epileptor model [43]:

$$\begin{array}{c} \begin{array}{c} \dot{v}=1+I_{app}-v^{3}-2v^{2}-z,\\ \dot{z}=\frac{\tau_{z}}{s}\left(c\left(v-v_{0}\right)+z\right), \end{array} \end{array}$$ (29)

where *v* and *z* represent the firing rate and the permittivity of a neuronal population, respectively. For this model we will work with the sets of parameters $P_{+},P_{0}$ and $P_{-}$ in Table 1.

**Table 1:** Different parameters for the reduced 2D Epileptor model in (29). For the set of parameters $P_{i}$, the system will display a limit cycle Γ_*i*_ of period *T*_*i*_.

	*τ*_*z*_	*v*_0_	*I*_*app*_	*c*	*s*	Lim. Cycle	Period
$\;P_{+}\;$	1/2857	-2	3.1	-4	-1	Γ_+_	*T*_+_ ≈ 2181.6
$P_{0}$	1/2857	-1.5	3.1	-16	-1	Γ_0_	*T*_0_ ≈ 695.7
$P_{-}$	1/2857	-0.1	3.1	2.4	1	Γ_−_	*T*_−_ ≈ 7333.3

**Fig 9:**
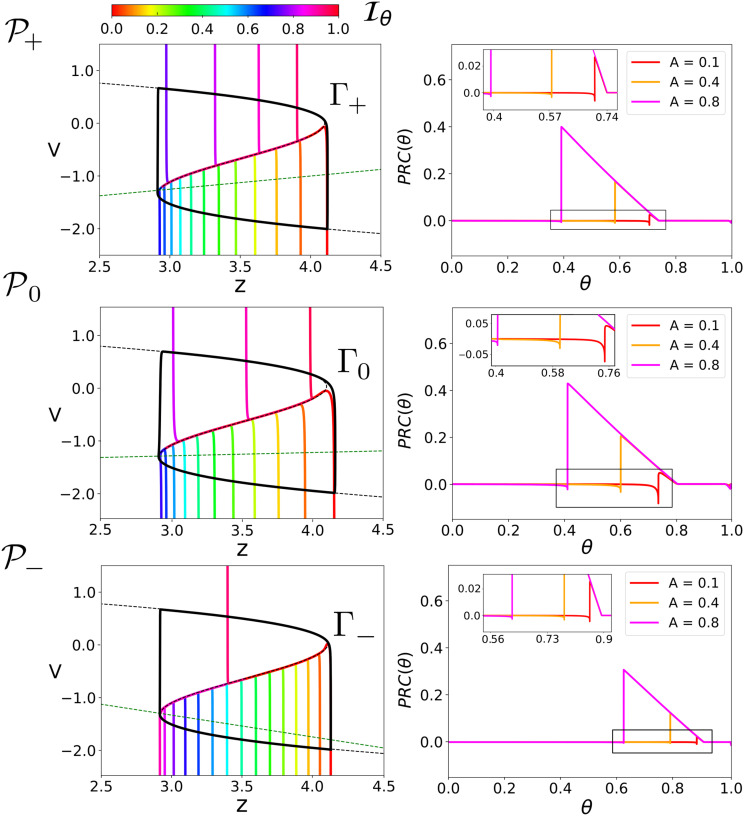
Isochrons and PRCs for the reduced epileptor. For the sets of parameters $P_{+},P_{0}$ and $P_{-}$ in Table 1 we show: Limit cycle Γ_+_, Γ_0_ and Γ_−_ and its isochrons $I_{\theta }$ (left). The phase response curves for pulses in the *v* direction for different values of *A* (right). For the three cases we plot 16 equispaced isochrons. Consistently with our previous analysis, since the monotonicity of the slow vector field does not change, the slope of isochrons does not change sign. For numerical details about the computation of both the isochrons and the PRCs see Materials and methods section.

Identically as the phenomenor, since the time constant for the *z* variable is small *τ*_*z*_ ≪ 1, and $\dot{v}=0$ describes a cubic curve, the three sets of parameters $P_{+},P_{0}$ and $P_{-}$ lead to relaxation oscillators denoted as Γ_+_, Γ_0_ and Γ_−_ respectively. The three different sets of parameters $P_{+},P_{0}$ and $P_{-}$ were chosen to illustrate the influence of the slow vector field on the response of perturbations of the system. Indeed, we denoted the parameters as $P_{+},P_{0}$ and $P_{-}$ because they set the nullcline to have positive, horizontal and negative slope, respectively. Fig 9 shows the isochrons and PRCs for the three sets of parameters $P_{+},P_{0}$ and $P_{-}$. Since the slow vector field of the reduced epileptor is monotonic in the fast variable *v*, the slope of the isochrons does not change sign for any of the considered cases, and again, it causes a prevalence of delays for perturbations of positive amplitude over points on the lower branch which is captured by the PRCs (see Fig 9). We remark the similarity between the computed PRCs in Fig 9 and the ones sketched in Fig 6.

### Response to perturbations

Next, we show how while the unperturbed behaviour of the cycles Γ_+_, Γ_0_ and Γ_−_ remains qualitatively identical, that is, they show relaxation oscillations, their response to the same train of pulses will be completely different. As we will argue, these remarkable differences can be explained by the different sets of parameters $P_{+},P_{0}$ and $P_{-}$ causing different slow vector fields for each cycle. Identically as in the phenomenor case, we consider a random train of pulses whose inter pulse interval follows a normal distribution of mean *T*_*s*_ and standard deviation *σ*, denoted by $N(T_{s},\sigma^{2})$ and compute the change of the seizure rate Δ for a train with $N(T_{s},0)$ and $N(T_{s},0.05T_{s})$.

**Fig 10:**
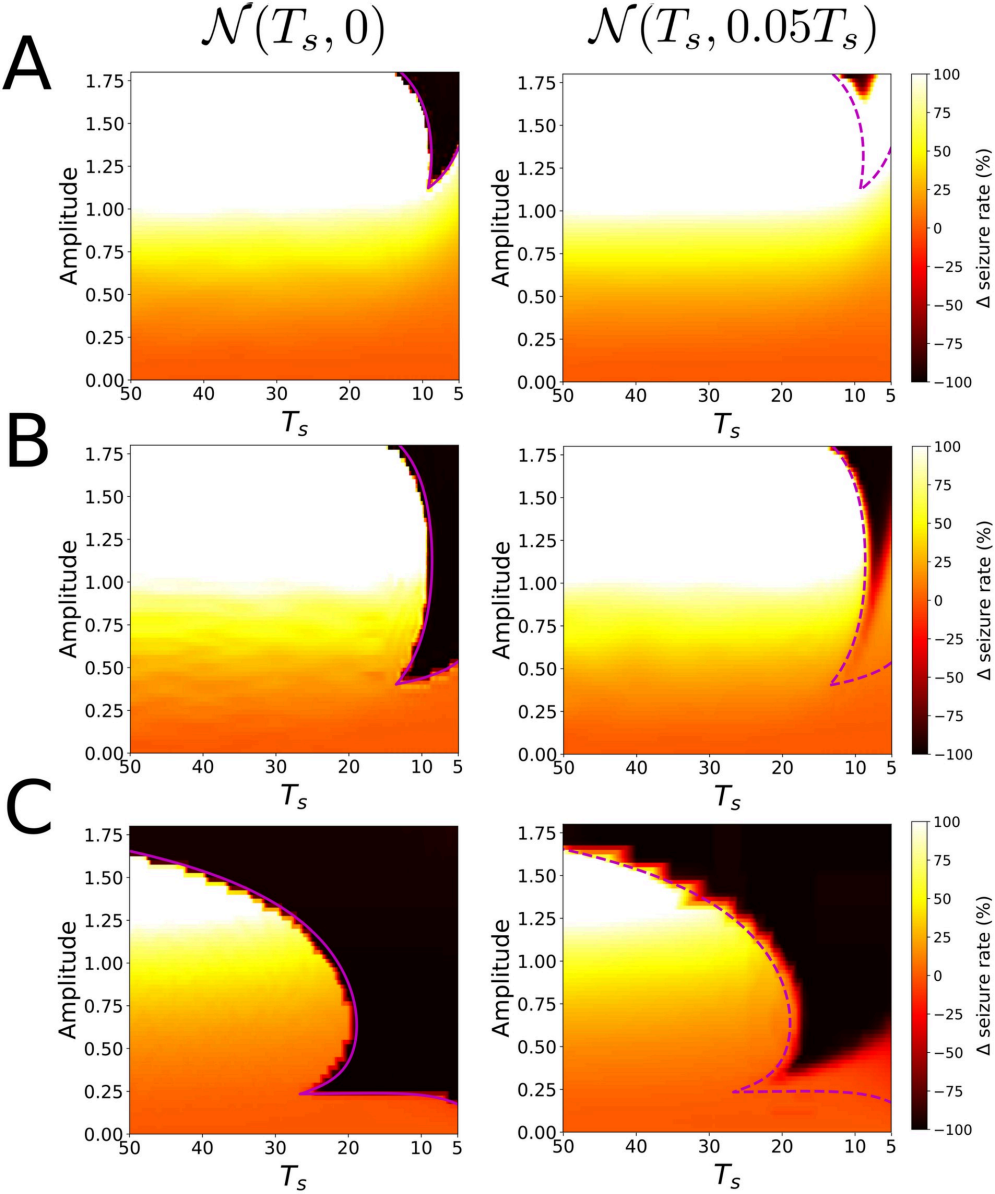
Response of perturbations for the reduced epileptor (29). We show the change in the seizure rate Δ for a random train of pulses whose mean inter impulse interval follows a normal distribution $N(T_{s},\sigma^{2})$ with a mean time *T*_*s*_ and standard deviation *σ*. Panels A, B, C correspond to the sets of parameters $P_{+},P_{0}$ and $P_{-}$ in Table 1. Left Figures correspond to the periodic case $N(T_{s},0)$ and right Figures to the random case $N(T_{s},0.05T_{s})$. Consistently with our theoretical analysis there is a direct correspondence between the mean distance between the lower branch and the slow nullcline and the minimal pulse amplitude *A* for which perturbations may lead to lock the system. Purple solid lines, bounding locking regimes, correspond to the bifurcations of the map (25). By drawing the same curve for the random case, we illustrate the resilience of locking states to noise. For the specific details about the numerical computation of this Figure, we refer the reader to Materials and Methods section.

The simulation results are summarized in Fig 10. Consistently with the theoretical results, we can see a direct correspondence between the mean distance between the lower branch and the slow nullcline and the appearance of areas suppressing the oscillation. For this reason, Γ_−_ locks for smaller amplitude values than for Γ_0_ and Γ_+_. Furthermore, although the bending of the isochrons is small and so are the corresponding delays Δ*θ*, because of its large *T* value (see Table 1), the range of *T*_*s*_ = −*T*Δ*θ* values for which Γ_−_ shows locking is even larger than for Γ_0_ and Γ_+_. We also remark the good agreement between the bifurcation curves of map (25) and the areas suppressing the transition to seizure.

Regarding the interpretation of the random perturbation train scenario, we can interpret results approximately by means of the results for the periodic perturbation scenario. Similarly as we argued in the phenomenor case (see Fig 8), the robustness of a given locking state to noise will depend on whether the critical value of $T_{s}^{*}=T\Delta \theta^{*}$, (where Δθ* corresponds with the maximal delay value of the PRC) is or not within the width *σ* of the distribution $t_{s}∼N\left(T_{s},\sigma^{2}\right)$. The higher the probability of occurrence of $t_{s}>T_{s}^{*}$ values, the likely is the system to switch to the upper branch. The differences in the resilience of the deterministic locking areas for Γ_+_, Γ_0_ and Γ_−_ in Fig 10, can be explained by the different values of the period for the 3 cycles (see Table 1). Despite the PRCs for the three cycles show a similar range of values for the delays Δ*θ*, the differences come when these delays are transformed in inter impulse intervals through *T*_*s*_ = *T*Δ*θ*. The shorter the period *T*, the smaller the critical $T_{s}^{*}=-T\Delta \theta^{*}$ value. Since in the three cases the *t*_*s*_ distributions have the same width, the smaller the critical $T_{s}^{*}$ value, the higher the probability of occurrence of $t_{s}>T_{s}^{*}$ values. As a consequence, the resilience of locking states for Γ_+_ and Γ_0_ is weaker than for Γ_−_ in which the distribution *T*_*s*_ = −*T*Δ*θ* is larger because of its larger period.

### Comparison between the phenomenor and the reduced epileptor

**Fig 11:**
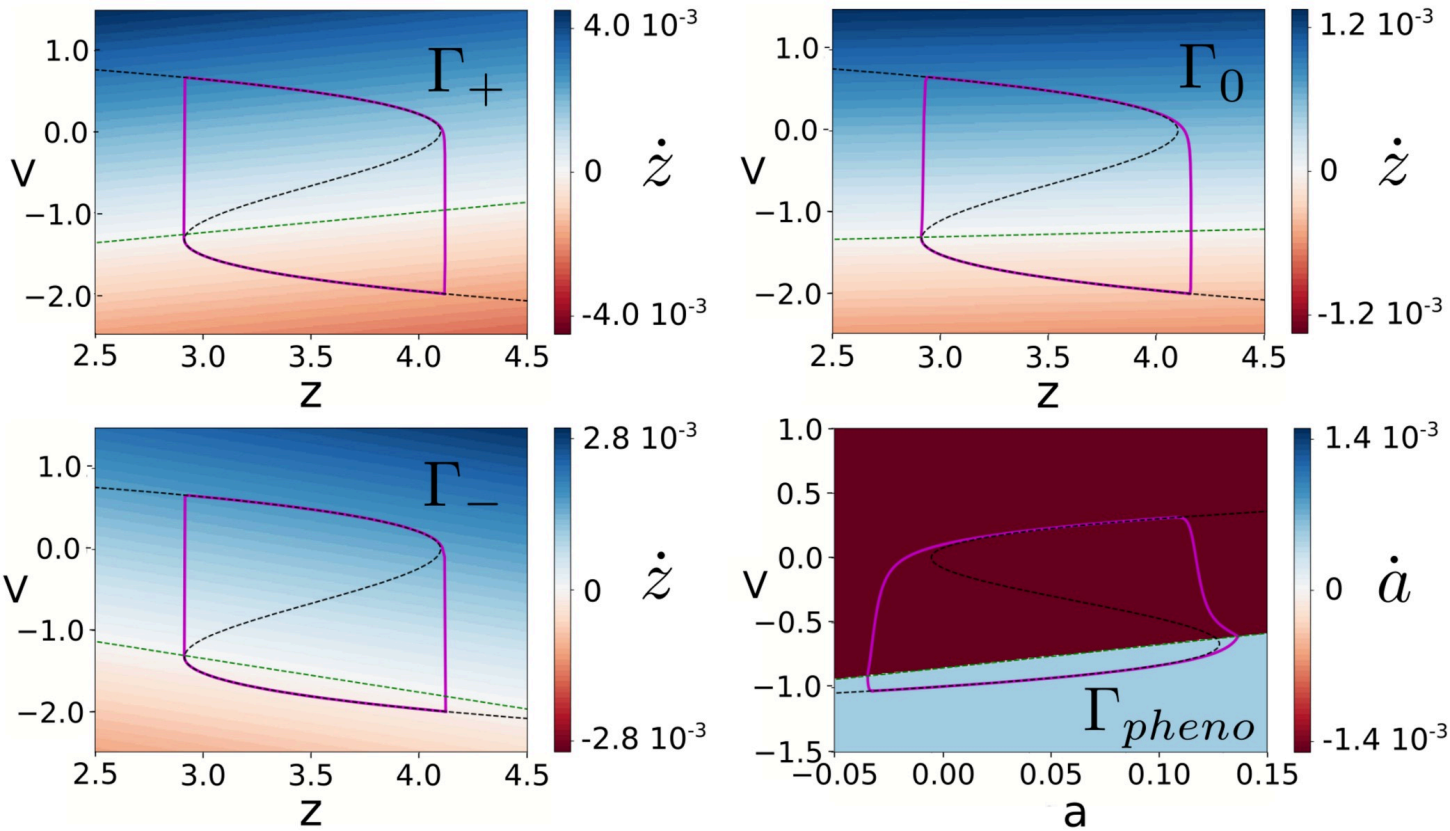
Slow vector field for the reduced epileptor and the phenomenor. Each cycle is depicted in purple, the v-nullcline in black and the slow nullcline in green. Notice that the direction of the slow variable in both models is flipped, and thus is also the motion over the cycles and the sign of the derivative of the slow vector field in the fast direction $g_{x}^{\prime }\left(x,y\right)$.

Although both the phenomenor in Eq (7) and the reduced epileptor in Eq (29) model seizure dynamics through relaxation oscillations, it is worth to mention the different role of the slow variable in the models. In the phenomenor the variable *a* describes the excitability of the tissue (the higher excitability, the more likely the spontaneous seizure initiation), whereas in the (both original and reduced) epileptor the *z* variable (dubbed as *permittivity*) has the opposite polarity: for its *low* values, the system switches to seizure as its only stable state. As a consequence, although the dynamical mechanism of the two models generate is virtually identical, the monotonicity of the slow vector field and the rotation direction over the cycle is flipped (see Fig 11). However, in both models, the motion and the tilt of the isochrons are related in such a way that the prevalent effect of positive voltage perturbations over the lower branch of the cycle is to slow-down the oscillations, or in particular to delay the seizures.

To further compare both models from a general mathematical perspective, we come back to the notation for a generic planar slow-fast system we defined in (1), where *g*(*x*, *y*) corresponds to the slow vector field and *x*, *y* to the fast and slow variables, respectively. The main differences between both models rely on their different time constant *τ* values and the specific slow vector field functions *g*(*x*, *y*). Because of the correspondence between *τ* ≪ 1 and *ϵ*, we expect the isochrons to be bounded in domains O(τ) (see Fig 4). However, from our analysis it also follows that the bending of the isochrons, although being contained in O(τ) domains, will be also determined by dependence of *g*(*x*, *y*) on the fast variable *x* between the perturbed and the base trajectories (see Eq (23)). To illustrate these role of *τ* and $g_{x}^{\prime }\left(x,y\right)$, let us compare Γ_*pheno*_ with Γ_−_. In both cases, the slow nullcline was near the lower branch, so we have a (qualitatively) similar geometry for both phase spaces. For this reason the response to perturbations was qualitatively similar in both cases (compare Figs 8 and 10C). However, the larger range of *T*_*s*_ values for which perturbations over Γ_*pheno*_ avoid seizure can be explained by both the larger *τ* and the strong change in the monotonicity of *g*(*x*, *y*) for the PE. The combination of both effects causes a larger bending of the isochrons and thus larger delays. Therefore, for (qualitatively) similar geometries, the differences in both the time constants values and in the strength of variations in the fast component of the slow-vector field have a substantial effect on the amplitude of the phase response of the system to inputs.

## Discussion

In this paper we applied a phase approach to analyse planar relaxation oscillators, motivated by models of epileptic dynamics. Indeed, the study of neural oscillators by means of the phase reduction has been extensively utilized in neuroscience from the level of single neurons to the network scale [28, 44–46]. In this work, the computation of isochrons and PRCs of the phenomenological seizure dynamics model introduced in [22] fully clarified the mechanism integrating the antagonistic potential effects of IEDs. Furthermore, the theoretical analysis of the phase response of a generic planar relaxation oscillator manifested the crucial role of the slow vector field on the geometry of their isochrons. Due to the direct link between isochrons and PRCs, we have been able to study the relationship between the slow vector field and the different response behaviour a planar oscillator can display depending on the amplitude and frequency of perturbations. For the cases considered, whereas the distance between the slow nullcline and the bottom branch of the cycle indicated the minimum value of amplitude values suppressing the original oscillation, the minimum value of PRCs (that is, the maximum delay) was related to the maximum interpulse intervals for which this locking mechanism holds. Furthermore, besides confirming our results, the study of variants of the reduced epileptor model [43] showed how vastly different responses to perturbations can be exhibited by models differing only in the slow-nullcline position, but possessing almost identical unperturbed behaviour, i.e. equivalent limit cycle oscillations, thus demonstrating the key role of the slow vector field in the response of perturbations for planar relaxation oscillators.

We acknowledge that due to the motivation by models of epilepsy, we showcased the theory only on a small set of example dynamical systems previously used for modelling the cyclical transition between an ictal and interictal state, which showed quite similar dynamics, including having one linear and one cubic nullcline, and a monotonous slow component of the flow field. A quick glance at other slow-fast relaxation oscillator models however suggests, that these properties are far from uncommon in many other models. Moreover, careful consideration of the theoretical arguments however shows, that the specific linear or cubic shape is indeed not crucial for the general observations to hold. Also, careful consideration of the theoretical arguments shows, that the monotonicity of slow vector field is firstly quite natural (the function needs to change from positive to negative values between the two stable branches of the stable manifold; it may likely do so just monotonically); and moreover not necessarily needed—if the change is not monotonic, the dependence of the PRC on the size of the perturbation just becomes more complicated, however the (sign of) the PRC is still given by the integral of the slow component along the recovery trajectory.

Another apparent limitation is that we focused on the effect of positive pulses acting on the bottom branch of the cycle. However, the approach straightforwardly extends to planar oscillators having more complex slow vector fields and to pulses of different sign applied either to the lower or higher branch. Indeed, we suggest that for a given slow vector field the applied geometrical approach is instrumental in providing an intuitive insight concerning the isochrons and therefore the PRCs. In that sense, our analysis extends previous results on PRCs and isochrons of planar relaxation oscillators beyond the weak and singular limit [39, 47]. Theoretically more interesting, while also more demanding, is the generalization to higher dimensional oscillators, providing richer geometrical structure of the flow, perturbations and trajectories. However, previous simulation-based results on the full Epileptor model [22] suggest that the potential dual effect of perturbations on oscillatory behaviour is preserved even in higher dimensions, although richer behaviour might show for other models or perturbation scenarios.

Regarding epilepsy, our results indicate the key influence of the slow vector field on the propensity for seizure emergence. We acknowledge our analysis relied on reduced planar models. However, we plan to make advantage of recent methodologies computing isochrons of high dimensional systems [48] to extend our approach to different high dimensional models as [17, 19–21, 49]. In general, the high dimensionality of these models permits to describe more accurately seizures initiation and termination [14, 50]. We believe the continuation of this line of research may provide an alternative vision to the questions these models approach. Furthermore, because of the usage of the phase variable and the determination of PRCs, we think this approach can also help to determine more accurately coupling functions for studies approaching epilepsy from the coupling of different oscillatory units [51].

Importantly, the quest for deeper and intuitive understanding of the effect of perturbation on epileptic network dynamics is not just an intriguing mathematical exercise, but an indispensable part of an important while difficult journey to understand the mechanisms of seizure initiation, and the possible ways to preclude this initiation by therapeutic stimulation interventions [52]. Of course, while the general conceptual insights are on their own relevant for general understanding the possible dynamical phenomena in response to perturbations, the observed role of the slow component of the field and in particular the nullcline suggests that any computational models of epilepsy dynamics should also attempt to reasonably approximate these aspects (and not only the unperturbed behaviour), if aspiring for providing relevant predictions concerning treatment protocols or just outcomes of endogenous perturbations and inter-regional interactions. This opens also the question of how to practically estimate these properties from experimental data, be it through stimulation protocols or purely observation data; this seems to be a natural avenue for obtaining more realistic models of epileptic dynamics.

In conclusion, we have outlined and carried out phase response analysis of planar relaxation oscillator models of epileptic dynamics that opens not only a path in epilepsy research with many interesting analytical, computational, experimental and potentially clinical implications, but also provides a framework applicable to gain insight in the plethora of other computational biology problems in which slow-fast relaxation oscillator models are pertinent.

## Materials and methods

This section contains some technical details concerning the numerical implementation of computations used to provide the presented results. Integration of ordinary differential equations was done using a 8th-order Runge-Kutta Fehlberg method (rk78) with a tolerance of 10^−14^.

### Counting of seizures

In this Section we explain how we generate the diagrams in Figs 2C, 8, and 10 showing the effect of perturbations on the transition to seizure for both the phenomenor (PE) and the reduced epileptor (RE). As we explain along the manuscript, both models describe epileptic dynamics through a relaxation oscillator of period *T* whose dynamics on the upper stable branch correspond to seizures. As for both models, the upper branch of the cycle terminates at the upper fold point $S_{+}^{f}$ (which in both cases corresponds to *v* = 0), we have proceeded this way: for each case we integrate the corresponding system for a time *t* ≫ *T* with a time step Δ*t* and apply a pulse of amplitude *A* in the *v* direction at equispaced *T*_*s*_ intervals. Each time the following condition is satisfied: *v*(*t* − Δ*t*) < 0 < *v*(*t*), we consider the system transitions to seizure. Finally, the change in seizure rate Δ is computed by dividing the number of seizures in the perturbed case by the number of seizures for the unperturbed case (which is 1 seizure per period). For the random case we proceed the same way just perturbing the system at intervals $t_{s}∼N\left(T_{s},\sigma^{2}\right)$.

From the adopted criteria for counting seizures it follows that very large perturbations might cause or constitute a seizure per se independently of the *T*_*s*_ value. For that reason, since we were interested in the relationship of both the amplitude *A* and the inter pulse interval *T*_*s*_ we limited the simulated amplitude in the above mentioned Figures to maximum of *A* = 0.8 for the PE and *A* = 1.8 for the RE (note that perturbations of *A* ≈ 1 for the PE and *A* ≈ 2 for the RE will cause seizures independently of the *T*_*s*_ value).

### Computation of isochrons

To compute isochrons of slow-fast systems, we assume we have an analytic vector field $\dot{z}=Z\left(z\right)$ having a *T*-periodic hyperbolic attracting limit cycle Γ which we parametrise by *γ*(*θ*) (see Eqs (9) and (10)). To find *γ*(*θ*), we construct a Poincaré section and use a Newton method to find a fixed point of the corresponding Poincaré map. By doing this, we obtain a point *z*_0_ ∈ Γ and the period *T*. Then, we integrate the system (9) with initial condition *z*(0) = *z*_0_ for a time *T* to obtain z(θT)≕γ(θ)forθ∈[0,1).

Next, we need to compute the linearisation *N*(*θ*) of the isochrons around Γ. To that aim, typically one solves a variational-like equation [53]. However, in slow-fast systems the cycle is strongly attracting (indeed, its characteristic multiplier is $O(e^{-k/\epsilon })$ with *k* > 0) [54]. For this reason, obtaining *N*(*θ*) via numerical integration requires to deal with very small numbers, so one needs high precision algorithms and large number of decimals.

As an alternative to numerical integration, we took advantage of the fact that ∇Θ(*z*) is perpendicular to the level curves of Θ(*z*), which indeed correspond to the isochrons. Therefore, we can use the infinitesimal PRC (iPRC), that is ∇Θ(*γ*(*θ*)), to compute *N*(*θ*) through the following equation [53]:

$$\begin{array}{c} \nabla \Theta \left(\gamma \left(t/T\right)\right)=\frac{N{\left(\theta \right)}^{⊥}}{T\langle N{\left(\theta \right)}^{⊥},Z\left(\gamma \left(\theta \right)\right)\rangle }, \end{array}$$ (30)

where *v*^⊥^ refers to a perpendicular vector to *v* and <⋅, ⋅> to the usual dot product. Instead of computing the iPRC ∇Θ(*γ*(*t*/*T*)) by integrating the adjoint equations (which also display numerical instabilities) we compute it by means of the procedure described in next subsection.

Finally, we globalise the isochrons via the backwards integration of *N*(*θ*) (we refer the reader to [53] for more details about the globalisation procedure).

### Computation of PRCs

The PRCs in this paper were computed using a continuation method. The computation of PRCs by direct integration of the perturbed trajectories, usually measures the phase shift over the maximum of a certain variable. That is, they require to integrate a relaxation time *T*_*rel*_ large enough so the perturbed trajectories reach the maximal values over the cycle. By contrast, as we now show, continuation methods just require the perturbed trajectories to reach a point on the cycle. Therefore, one needs to integrate a shorter time *T*_*rel*_. Specifically in slow-fast systems, in which the periods of the system are large, the usage of continuation methods saves a lot of computational effort. To compute PRCs, we have used the continuation method introduced in [55], which we now briefly review for the sake of completeness.

A pulse acting on a point *z* = *γ*(*θ*) ∈ Γ will displace the trajectory to $\bar{z}=z+A$. Then, after a time *T*_*rel*_ large enough, the trajectory will be again on the limit cycle but with another phase $\bar{\theta }$. Mathematically

$$\begin{array}{c} F_{A}\left(\gamma \left(\theta \right)\right)=\gamma \left(f_{A}\left(\theta \right)\right), \end{array}$$ (31)

where $F_{A}\left(z\right)=\phi_{T_{rel}}\left(z+A\right)$, and $f_{A}\left(\theta \right)=\bar{\theta }$. Then the PRC is *PRC*(*θ*, *A*) = *f*_*A*_(*θ*) − (*θ* + *T*_*rel*_/*T*).

The idea of the method is to obtain *f*_*A*_(*θ*) by solving Eq (31). To that aim, one can use the following algorithm which computes the PRC for a perturbation of amplitude *A* by means of a Newton method. The computation of PRCs via continuation is achieved using the computed PRC as an initial seed for computing the PRC for a new amplitude *A*′ = *A* + Δ_*A*_. Given the parameterization of the limit cycle *γ*(*θ*), and *f*_*A*_(*θ*) an approximate solution of Eq (31), we perform the following operations:

1. Compute *E*(*θ*) = *F*_*A*_(*γ*(*θ*)) − *γ*(*f*_*A*_(*θ*)).
2. Compute ∂_*θ*_ *γ*(*f*_*A*_(*θ*)) = *TZ*(*γ*(*f*_*A*_(*θ*))).
3. Compute $\Delta f_{A}=\frac{<\partial_{\theta }\gamma \left(f_{A}\left(\theta \right)\right),E\left(\theta \right)>}{<\partial_{\theta }\gamma \left(f_{A}\left(\theta \right)\right),\partial_{\theta }\left(f_{A}\left(\theta \right)\right)>}$.
4. Set *f*_*A*_(*θ*) ← *f*_*A*_(*θ*) + Δ*f*_*A*_(*θ*).
5. Repeat steps 1-4 until the error *E* is smaller than the established tolerance. Then *PRC*(*A*, *θ*) = *f*_*A*_(*θ*) − (*θ* + *T*_*rel*_/*T*).

We refer the reader to [55] for the implementation of this methodology for not pulsatile perturbations. To compute the iPRC by means of this algorithm one has to consider perturbations of *A* small and *f*_*A*_(*θ*) = *θ* + *T*_*rel*_/*T* as initial seed. Then, ∇Θ(*γ*(*θ*)) = *PRC*(*A*, *θ*)/*A*.
